# Advanced CT acquisition protocol with a third-generation dual-source CT scanner and iterative reconstruction technique for comprehensive prosthetic heart valve assessment

**DOI:** 10.1007/s00330-017-5163-7

**Published:** 2017-12-12

**Authors:** Marguerite E. Faure, Laurens E. Swart, Marcel L. Dijkshoorn, Jos A. Bekkers, Marcel van Straten, Koen Nieman, Paul M. Parizel, Gabriel P. Krestin, Ricardo P. J. Budde

**Affiliations:** 1 Department of Radiology, University Hospital of Antwerp, Wilrijkstraat, 10 2650 Edegem, Belgium; 2 Department of Radiology & Nuclear Medicine, Erasmus MC, Rotterdam, The Netherlands; 3 Department of Cardiology, Erasmus MC, Rotterdam, The Netherlands; 4 Department of Cardiothoracic surgery, Erasmus MC, Rotterdam, The Netherlands

**Keywords:** Prosthetic heart valve, Image quality, Cardiac imaging, Computed tomography, Radiation dose

## Abstract

**Objectives:**

Multidetector CT (MDCT) is a valuable tool for functional prosthetic heart valve (PHV) assessment. However, radiation exposure remains a concern. We assessed a novel CT-acquisition protocol for comprehensive PHV evaluation at limited dose.

**Methods:**

Patients with a PHV were scanned using a third-generation dual-source CT scanner (DSCT) and iterative reconstruction technique (IR). Three acquisitions were obtained: a non-enhanced scan; a contrast-enhanced, ECG-triggered, arterial CT angiography (CTA) scan with reconstructions at each 5 % of the R-R interval; and a delayed high-pitch CTA of the entire chest. Image quality was scored on a five-point scale. Radiation dose was obtained from the reported CT dose index (CTDI) and dose length product (DLP).

**Results:**

We analysed 43 CT examinations. Mean image quality score was 4.1±1.4, 4.7±0.5 and 4.2±0.6 for the non-contrast-enhanced, arterial and delayed acquisitions, respectively, with a total mean image quality of 4.3±0.7. Mean image quality for leaflet motion was 3.9±1.4. Mean DLP was 28.2±17.1, 457.3±168.6 and 68.5±47.2 mGy.cm for the non-contrast-enhanced (n=40), arterial (n=43) and delayed acquisition (n=43), respectively. The mean total DLP was 569±208 mGy.cm and mean total radiation dose was 8.3±3.0 mSv (n=43).

**Conclusion:**

Comprehensive assessment of PHVs is possible using DSCT and IR at moderate radiation dose.

***Key points*:**

*• Prosthetic heart valve dysfunction is a potentially life-threatening condition.*

*• Dual-source CT can adequately assess valve leaflet motion and anatomy.*

*• We assessed a comprehensive protocol with three acquisitions for PHV evaluation.*

*• This protocol is associated with good image quality and limited dose.*

## Introduction

Prosthetic heart valve (PHV) dysfunction is an uncommon but important and potentially life-threatening complication after PHV implantation [1]. In daily practice, the most commonly used imaging modality for evaluating PHVs is transthoracic echocardiography (TTE) [2]. TTE has known limitations such as a high interobserver variability, the possibility of poor acoustic windows and acoustic shadowing. Hence, evaluation of PHV abnormalities often also requires transoesophageal echocardiography (TEE), which is a semi-invasive procedure associated with certain risks and complications, and is also affected by some of the TTE limitations, albeit to a lesser extent [3].

More recently, multidetector computed tomography (MDCT) has been shown to provide complementary information for PHV assessment and is useful to identify the cause of PHV dysfunction, including obstructive masses (such as pannus and thrombus), prosthesis-patient mismatch, paravalvular regurgitation, as well as infective endocarditis and its complications [4, 5]. Most often, a retrospectively ECG-gated CT angiography (CTA) acquisition is used to assess PHV dysfunction since it allows for reconstructions at each 5–10 % of the R-R interval needed to dynamically assess valve leaflet motion and anatomy. Such acquisitions are associated with a relatively high radiation dose as reported in multiple studies (mean 11.6 mSV [interquartile range (IQR) 10.8–14.4], 11.8 mSV [IQR 11.2–12.8] and 18.8 ±3.8 mSV [IQR 6, 7, 8]). Ideally, a CT acquisition protocol for PHV assessment would not only include a dynamic CTA but also a non-enhanced scan of the valve region to assess calcifications and suture pledgets, as well as a delayed phase of the entire chest for possible abscesses, septic pulmonary emboli and overall thoracic anatomy, which would increase radiation dose even further [9]. Third-generation dual-source scanners allow for several dose-reduction strategies that are advantageous for PHV assessment. We examined whether a novel, moderate radiation dose three-phase CT acquisition protocol for third-generation dual-source CT (DSCT) allows comprehensive ECG-triggered PHV assessment with both static and dynamic reconstructions to assess the PHV region as well as the entire chest.

## Materials and methods

### Patients

All consecutive patients with a PHV that underwent a CT can with this specific acquisition protocol in our department between December 2014 and November 2016 were included. The acquisition was a part of the routine clinical workup, and data were gathered retrospectively. No additional acquisitions were made specifically for this study. Patient data were retrieved from the electronic patient files. A waiver for retrospective evaluation of the data was obtained from the medical ethics committee.

### CT scan protocol

**Fig. 1:**
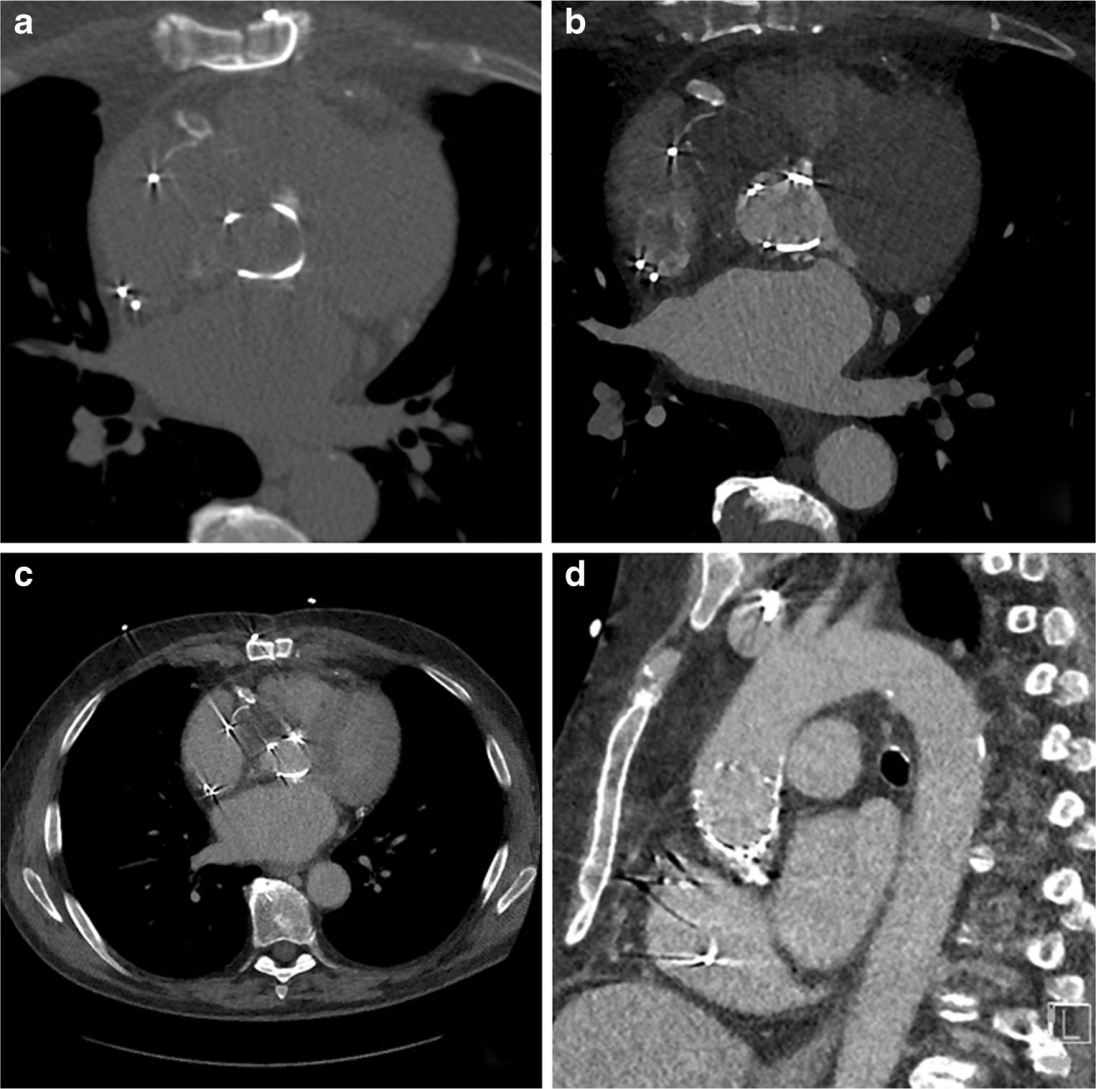
Image acquisition includes three sequential acquisitions: A non-contrast-enhanced scan (**A**); a contrast-enhanced arterial CT angiography (CTA) with reconstructions at each 5 % of the R-R interval (**B**); and a delayed high pitch CTA of the entire chest (**C**), which allows for evaluation of the complete thoracic aorta as shown on this sagittal view (**D**)

Image acquisition was performed on a third-generation DSCT (SOMATOM Force, Siemens, Erlangen, Germany). Image acquisition included three consecutive scans (Table 1, Fig. 1).

**Table 1:** Prosthetic heart valve scan protocol

Prosthetic valve protocol	Non-enhanced scan	Contrast-enhanced, ECG-triggered, CTA	Delayed high-pitch CTA of the entire chest
Scan start	2 cm above valve	Center of scan centered	2 cm above arch
Scan end	2 cm below valve	On valve	Bottom of heart
Scan length	Variable	Fixed 14 cm (3 stacks)	Variable
Scan type	Prospective step and shoot	Prospective step and shoot	Prospective high pitch spiral flash
Collimation	Adapted to scan length	192 x 0.6 mm	192 x 0.6 mm
Rotation time (ms)	250	250	250
Pitch	n.a.	n.a.	3.2
Feed/rot (mm)	variable	48	184
Reference mAs/rot	80	180	180
mA modulation	Yes, CARE DOSE 4D	Yes, CARE DOSE 4D	Yes, CARE DOSE 4D
Reference kVp	120	120	120
kVp modulation	CARE kV semi 120	CARE kV semi 120	CARE kV semi 120
Tissue of interest	Non-contrast	Vascular	Vascular
Beta blocker/Nitrates	no	no	no
ECG padding	45 %–45 %	0–1,500 ms	n.a.
ECG pulsing	off	50–500 ms	n.a.
Contrast	no	yes	yes
Type		Iodixanol*320mgl/ml	
Bolus		80 ml at 5 ml/s	
Chaser (30 % mix ratio)		20 ml at 3.5 ml/s	
delay		Bolustracking	70 s post-injection
Slice width (mm)	3.0	0.75	0.75
Slice increment (mm)	3.0	0.4	0.4
Kernel	Qr36	Bv40	Bv40
ADMIRE strength	3	3	3
ECG phase %	45 %	10–100 % at 5 % interval	30 % at trachial carina level
ECG phase ms	n.a.	Best systolic ms phase by computer	n.a.

*Visipaque, GE Healthcare

*CTA* CT angiography

First, a non-enhanced prospectively ECG-triggered scan of the PHV region alone was performed with the following acquisition parameters: fixed tube voltage 120 kV, reference effective tube load 80 mAs, collimation adapted to fit scan length, and gantry rotation time 250 ms. Data were acquired and reconstructed (slice thickness 3 mm, increment 3 mm, Qr36 kernel) for the 45 % phase of the R-R interval with iterative reconstruction (IR) (Admire Level 3, Siemens).

**Fig. 2:**
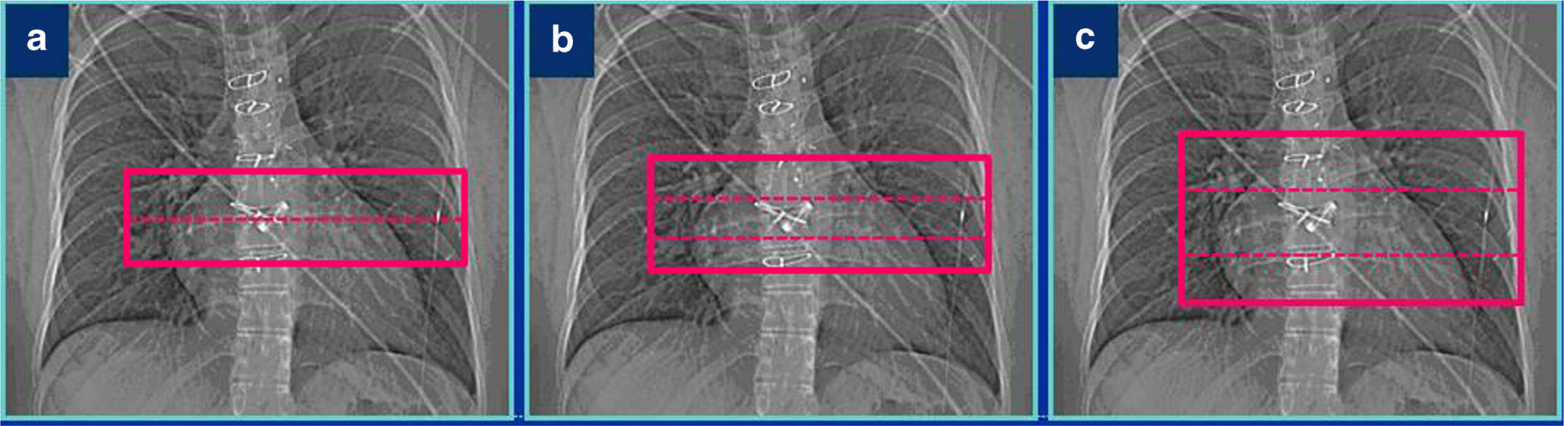
CT angiography (CTA) acquisition: Effect of scan range length in CTA: With prospective protocols in third-generation dual-source CTA the collimation is adapted to the set scan length. In regular cardiac examinations, this helps to avoid overscanning and results in an interpatient variability of the coverage per stack. When scanning heart valves full heart coverage is not always necessary and a shorter range may be sufficient. Special care should be taken in planning the scan range, however, so the largest possible *z* coverage per stack is obtained to avoid stack artefacts at the valve level. **A** A short scan range consisting of two stacks will result in potential stack artefacts through the valve. **B** A slightly longer scan range will result in three thin collimate stacks with the valve in the middle of the mid stack. Heart motion and respiratory variability might still move the valve into the potential stack artefacts. **C** Opening the scan length to exactly 14 cm will result in three stacks with maximum stack coverage ensuring valve images without ECG stack artefacts

**Fig. 3:**
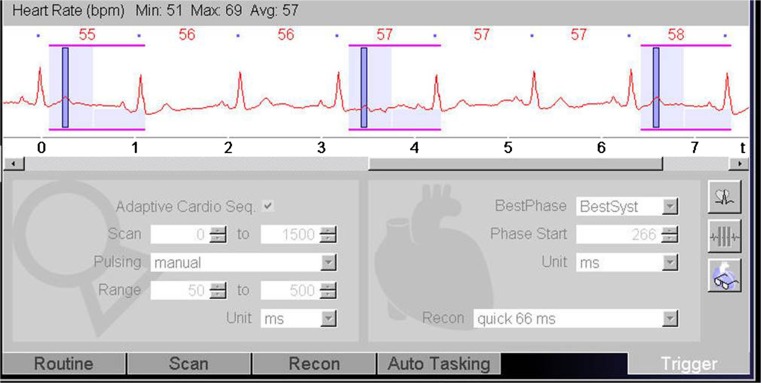
Screenshot from scanner console: The ECG-trigger in this CTA acquisition is based on absolute millisecond (ms) time instead of a relative (%) RR-trigger. A ‘scan-on–scan-off’ padding setting from 0–1,500 ms with 50–500 ms ECG-pulsing results in a scan acquisition window that is guaranteed to provide the fastest onset of radiation after an R-peak (minimum delay is ±100 ms, which is 10 % at 60 bpm). Radiation is maintained for 1,500 ms (which is a full heart cycle at 40 bpm) or is cut off when the next R-peak occurs. This means that independent of the heart rate, a full heart cycle (starting from 100 ms) is guaranteed

Secondly, a prospectively ECG-triggered wide-pulsing window sequential CT angiography (CTA) was performed with the following parameters: fixed tube voltage of 120 kV, reference effective tube load 180 mAs, collimation 192 × 0.6 mm and gantry rotation time of 250 ms. This prospectively ECG-triggered sequential scan was made with three, five or seven stacks. Care was taken to centre the middle stack on the prosthetic valve to avoid any stack artefacts at the level of the valve. For three stacks, the scan length in the z-axis was set at 14 cm, to allow for a maximum coverage per stack in the longitudinal direction (Figs. 2 and 3). Reducing the scan length will reduce the coverage of each of the three stacks evenly until the coverage can be accommodated by two stacks, thereby increasing the risk of stack artefacts. For this CTA, we used an ECG-trigger based on an absolute delay time after an R-peak instead of a relative (%) R-R trigger delay. Irrespective of heart rate, a fixed ‘scan-on–scan-off’ padding setting from 0–1,500 ms with 50–500 ms ECG-pulsing was used, which resulted in a scan acquisition window that is guaranteed to provide the fastest onset of radiation after an R-peak. After 500 ms, tube current (and thus radiation) decreased from 100 % to 20 % of the set level. Radiation was then maintained for 1,500 ms (which is a full heart cycle at 40 bpm) or cut off when the next R-peak occurred. Reconstructions were made at each 5 % of the R-R interval, with a slice thickness of 0.75 mm, an increment of 0.4 mm and Bv40 kernel (Figs. 2 and 3). An iterative reconstruction technique was used (Admire level 3, Siemens).

Thirdly and finally, a low-dose high-pitch CTA of the entire chest was performed, which was triggered in such a way that the scan reaches the trachial carina level at 30 % of the R-R interval. The scan was made with the following parameters: automatically selected tube voltage, reference effective tube load 180 mAs at 120 kV, collimation 192 × 0.6 mm, and a pitch of 3.2. Reconstructions were made with an increment of 0.4 mm, a slice thickness of 0.75 mm in Bv40 kernel and iterative reconstruction (Admire level 3, Siemens).

A single biphasic contrast administration protocol was used in which a total amount of 100 ml of contrast was given with an Iodine concentration of 320 mg/ml (Visipaque 320®, GE Healthcare, Cork, Ireland). First, 80 ml of contrast was injected at a rate of 5.0 ml/s, followed by 20 ml of contrast at 3.5 ml/s and a saline flush of 25 ml at 2.5 ml/s. A region of interest was defined in the ascending aorta and data acquisition was initialized when the threshold of 100 HU was reached. The delayed acquisition was obtained 70 s after contrast injection. No beta blockers or nitroglycerin were administered since they may be contraindicated in patients with valvular pathology.

Radiation dose was obtained from the reported CT dose index (CTDI) and dose length product (DLP) and converted to an effective dose, using a conversion factor of 0.0145 mSv/mGy*cm [10]. Heart rate during the acquisitions was obtained from the automatically generated information stored with the DICOM images.

### Image analysis

Assessment of the CT examinations was performed on a dedicated workstation (Philips Intellispace Portal, v6.0.3.12200). Using multiplanar reformation, images were reconstructed in plane with the PHV and perpendicular to the PHV and valve leaflets. Cine mode was used to dynamically evaluate PHV leaflet motion in all reconstructed phases of the RR interval. Image quality was scored separately for each of the three acquisitions in the same way, using a 5-point scale, adapted from a 4-point scoring system used in a previous study by our group [11]. The criteria for the different scores were formulated as follows: 1 – no discernible detail, no diagnostic information can be obtained; 2 – only limited visualization of the region of interest, no accurate measurements can be made; 3 – image quality is moderate, measurements and diagnosis are possible; 4 – image quality is good, no diagnostic or measurement problems; 5 – image quality is excellent. Furthermore, image quality of PHV leaflet motion was scored in the same way: 1 – leaflet motion not assessable; 2 – leaflet motion assessable, no reliable measurements can be made; 3 – leaflet motion assessable with the ability to measure opening and closing angles; 4 – good image quality of the leaflet motion; 5 – excellent assessment of leaflet motion possible. Scoring was performed by two radiologists (RB and MF) who had 10 and 2 years of experience with cardiac CT, respectively. The best image quality phase for assessing PHV could be variable among patients. Therefore, for each acquisition, the best cardiac phase was selected for scoring image quality. All images were clinically assessed for PHV-related pathology such as paravalvular leakage, thrombus and/or pannus formation, left ventricular outflow tract (LVOT) obstruction and abscesses.

### Data analysis

Descriptive statistics were used to analyse the study data. Mean values and standard deviations were calculated. Interobserver variability was analysed by weighted kappa statistics based on Cohen’s statistic.

## Results

### Patient characteristics

A total of 43 CT examinations acquired in 41 patients were included (mean age 60±23 years, 22 males). One patient who was imaged on three separate occasions was included three times. PHVs were positioned in the aortic (n=35), mitral (n=6) and pulmonary position (n=3). One patient had two PHVs. Of all 44 PHVs examined, 21 were mechanical valves and 23 were biological valves (Table 2). The main indications for referral were suspicion or follow-up of endocarditis (n=22), increased pressure gradient or suspicion of valve obstruction (n=10), suspicion or follow-up of paravalvular leakage (n=4). Other indications were follow-up after coarctation repair (n=2), after tetralogy of Fallot repair (n=1), follow-up of Marfan’s disease (n=1), after a Bentall procedure (n=1), after closure of a pseudo-aneurysm (n=1) and newly diagnosed pleural effusion(n=1).

**Table 2:** Patient characteristics

Gender (n=41)	22 male	19 female	
Age, years (mean) (n=41)	60±23		
PHV position (n=44)	Aortic: 35	Mitral: 6	Pulmonary: 3
PHV type (n=44)	Mechanical: 21	Biological: 12	TAVI: 11
	St-Jude: 19	Perimount: 6	Corevalve: 6
	Carbomedics: 1	Sorin: 1	Lotus: 3
	Björk-Shiley: 1	Perigon: 1	Sapien: 2
		Homograft: 4	

*PHV* prosthetic heart valve

### CT parameters

The non-enhanced acquisition was not performed in three patients. In all other patients (n=40), the three acquisitions were obtained. Mean heart rate during scanning was 70±19 bpm for the non-enhanced scan, 69±13 bpm for the ECG-triggered CTA and 75±25 bpm for the high-pitch CTA. Heart rate variability was calculated for the different stacks of the non-contrast-enhanced scan and the CTA, using 1 standard deviation of the mean heart rate. This was 2.3 bpm for the non-contrast-enhanced scan and 2.8 bpm for the CTA. The maximum heart rate variability was 24 bpm for the non-enhanced scan and 32.3 bpm for the CTA. The number of stacks used was 1 (n=10), 2 (n=23), 3 (n=5) and 4 (n=2) for the non-enhanced scan and 3 (n=41), 5 (n=1) and 7 (n=1) for the ECG-triggered CTA. Stack artefacts were seen in one out of 30 non-contrast enhanced CT scans (ten non-contrast scans were scanned with only one stack, so artefacts could not occur) and in two out of 43 ECG-triggered CTAs. In all these examinations, the artefacts did not appear at the valve position. In the delayed acquisition with automatic tube voltage selection, in one of 43 scans (2 %) the reference voltage of 120 kV was chosen, in three scans (7 %) the tube voltage was 100 kV, in four scans (9 %) 70 kV, in nine scans (21 %) 90 kV and in most of the scans (26 scans, 61 %) 80 kV was chosen.

### Radiation dose

Mean CTDI was 3.13±1.28, 30.92±9.6 and 2.31±0.94 mGy for the non-enhanced (n=40), CTA (n=43) and delayed acquisitions (n=43), respectively. Mean DLP was 28.2±17.1, 457.3±168.6 and 68.5±47.2 mGy.cm for the non-contrast-enhanced (n=40), arterial (n=43) and delayed acquisition (n=43), respectively. The mean total DLP was 569±208 mGy.cm. Mean total radiation dose was 8.3±3.0 mSv (range 3–17, n=43) (Table 3). In one study, the arterial CTA had seven stacks, with a wider scan range due to coronary artery bypass grafting (CABG), which resulted in a total dose of 16.7 mSv. In two studies, the abdomen was included in the high-pitch acquisition with a total radiation dose of 16.8 mSv and 6.7 mSv, respectively. Furthermore, one study had an additional high-pitch acquisition with a total radiation dose of 12.7 mSv.

**Table 3:** Radiation dose overview of the different acquisitions

	Non-contrast CT scan (mean±SD)	Arterial CT scan (mean±SD)	Delayed CT scan (mean±SD)
CTDI (mGy)	3.13±1.27	31.01±9.57	2.32±0.93
DLP (mGycm)	28.1±16.9	458.3±166.7	68.7±46.7
Dose (mSv)	0.41±0.23	6.65±2.42	1.00±0.68

*CTDI* CT dose index, *DLP* dose length product

### Image quality scores

**Fig. 4:**
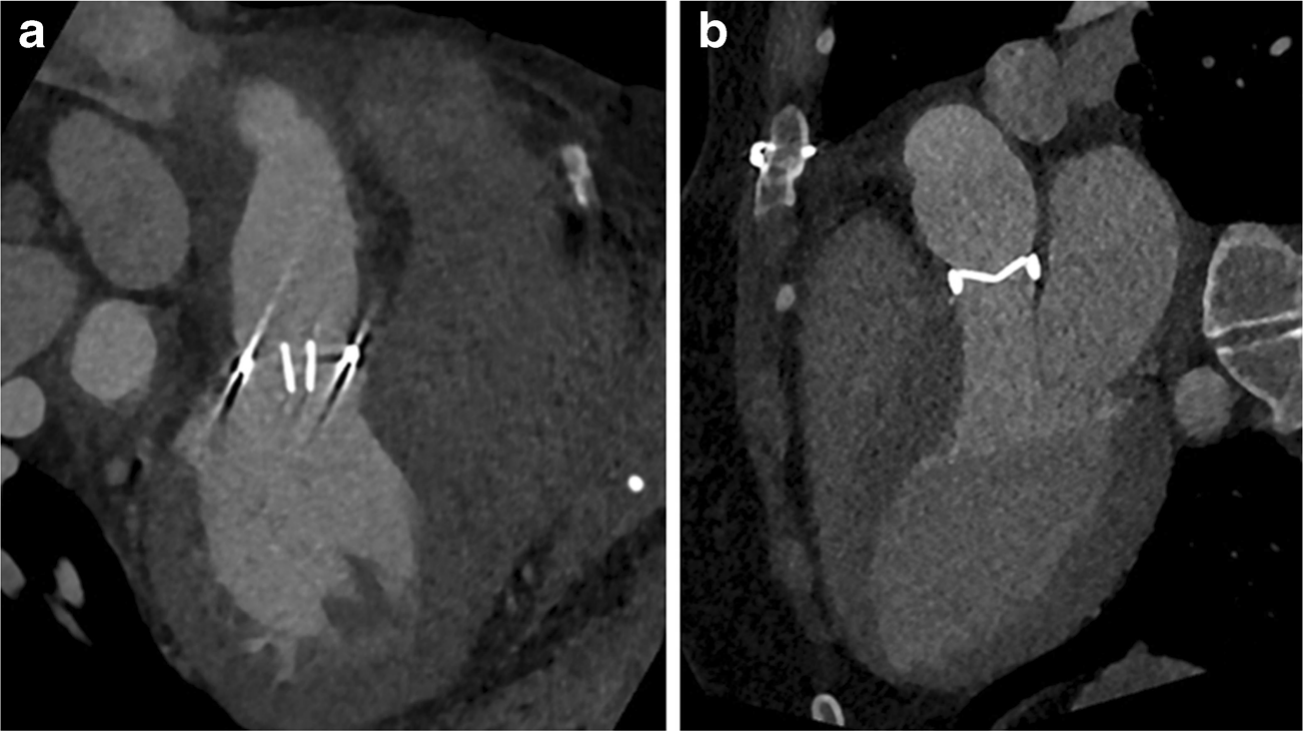
Image quality. Example of CT angiography (CTA) reconstruction in systole and diastole showing excellent image quality of a bi-leaflet prosthetic heart valve in the aortic position with valve in open (**A**) and closed (**B**) position

The mean image quality score was 4.1±1.4, 4.7±0.5 and 4.2±0.6 for the non-enhanced, arterial CTA and delayed acquisition, respectively, with a total mean image quality of 4.3±0.7 (Fig. 4). Mean image quality for leaflet motion was 3.9±1.4. Only one non-enhanced scan had a score of 1 and was not interpretable due to the presence of a cobalt- and chromium-containing mechanical heart valve (Björk-Shiley valve), which gives substantial artefacts. Another non-enhanced scan had a score of 2 with limited image quality due to a too short scan length with incomplete visualization of the heart. Overall, 124 out of 126 (98 %) acquisitions (40 non-contrast-enhanced, 43 CTA and 43 delayed acquisitions) had an image quality score of ≥ 3. Regarding leaflet motion, four scans had a score of 1 and were non-diagnostic and four had a score of 2 with only poor image quality. These were five transcatheter valves (four Corevalves and one Sapien valve) and three conventional biological valves. Thirty-four scans had an image quality score of ≥ 3 (81 %) with respect to leaflet motion.

**Fig. 5:**
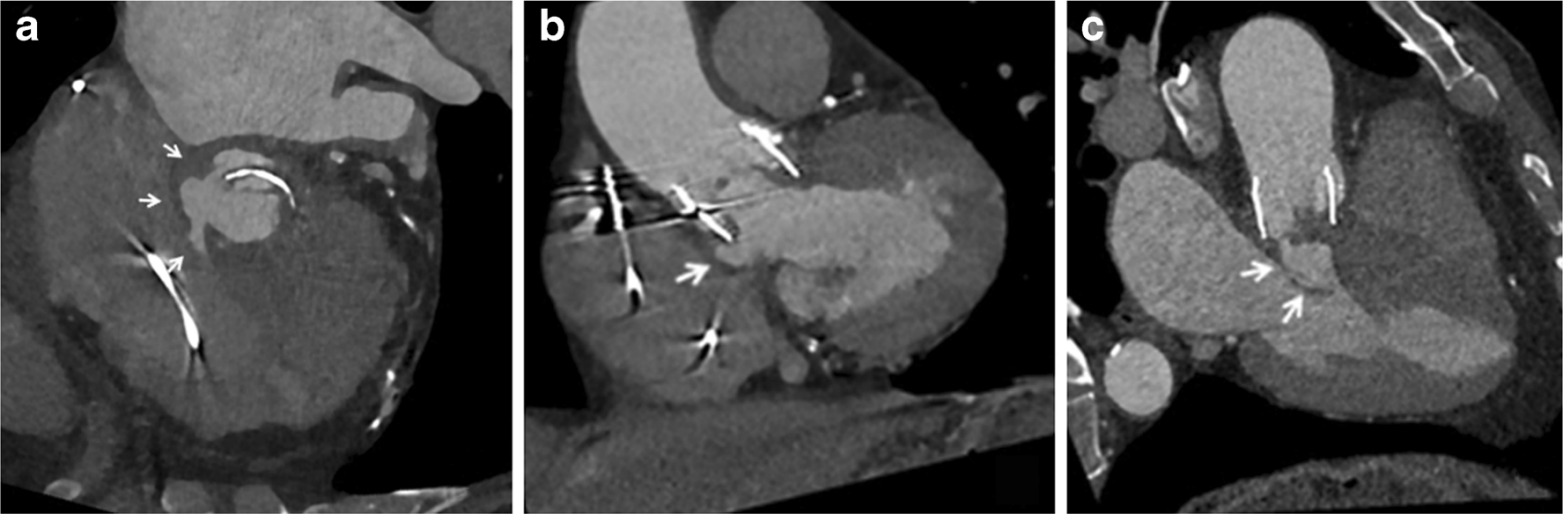
Patient with aortic prosthetic heart valve (PHV) (TAVR, type Lotus valve) with suspected endocarditis. CT shows PVL and false aneurysm formation. **A**: Axial contrast-enhanced ECG-triggered CT image shows paravalvular leakage (arrows). **B** (coronal) and **C** (sagittal) contrast-enhanced ECG-triggered CT images nicely show the PHV in the aortic position and the false aneurysm formation underneath (arrows).

Out of 43 examinations, 16 showed no PHV-related abnormalities. Five examinations revealed a false aneurysm at the aortic root. Three examinations showed a clear paravalvular leakage (PVL) and four had possible PVL (Fig. 5). Five examinations showed pannus or thrombus under the PHV and six examinations showed vegetations/structures inside the PHV. Abscess formation was found in one examination. Five examinations showed a cavity under the PHV. Three examinations revealed thrombus in the left atrial appendage. On the other hand, two patients had suboptimal filling of the left atrial appendage at the CTA, but thrombus could be excluded with the delayed scan.

### Interobserver agreement

The weighted kappa value for the image quality scores of both observers was 0.74 for the non-contrast-enhanced scan, 0.87 for the CTA and 0.80 for the delayed high-pitch scan. For scoring image quality of leaflet motion the kappa value was 0.80. This means there was a good interobserver agreement for the non-enhanced scan and a good-to-excellent agreement for the other acquisitions and for scoring image quality of leaflet motion.

## Discussion

**Fig. 6:**
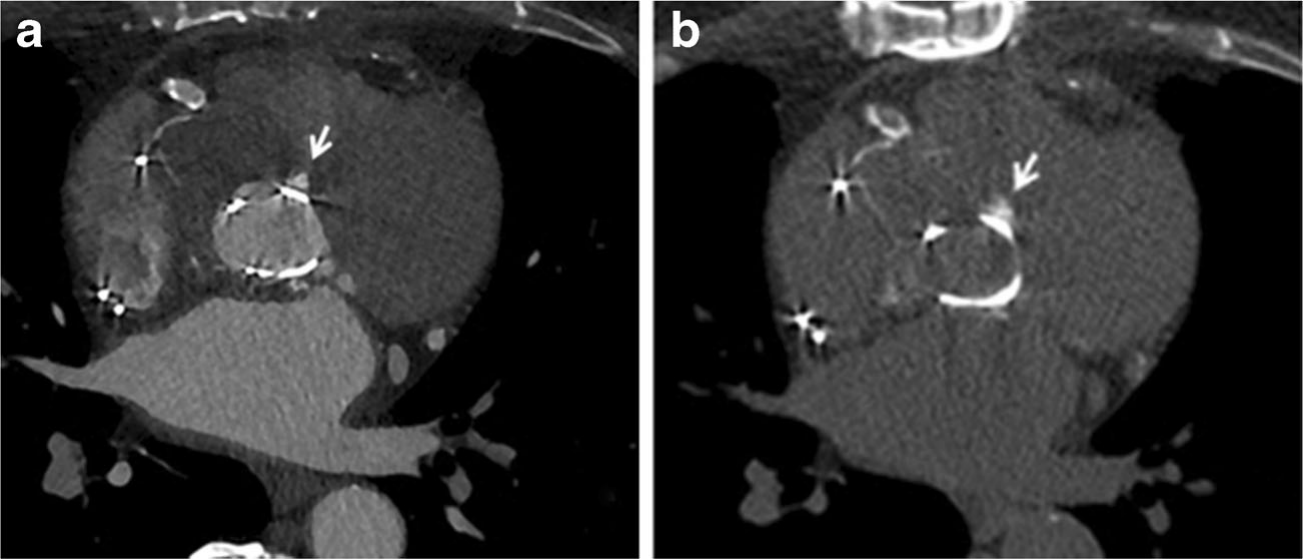
The non-contrast scan can identify sutures with polytetrafluorethylene felt pledgets. These pledgets can be isodense to contrast and mimic paravalvular leakage (PVL) on CT angiography (CTA) (**A**) and a non-contrast scan (**B**) can thus be helpful to differentiate between the two. Arrow indicates possible PVL on the CTA and confirms the hyperdense nature of the pledget on the non-contrast scan

Several studies have demonstrated the potential and incremental value of MDCT in patients with suspected PHV dysfunction [6–8]. However, these studies used retrospective ECG-gating CT angiography with a relatively high mean radiation dose of well over 10 mSv for a single arterial phase acquisition only. Utilizing the capabilities of a modern dual-source scanner, we devised a comprehensive multiphase acquisition protocol for PHV assessment that aims to reduce radiation dose but at the same time increase coverage and assessment potential in multiple phases. Our study shows that this acquisition protocol was associated with good overall image quality and a moderate radiation dose of 8.3 ±3.0 mSv while making three consecutive acquisitions. Furthermore, leaflet motion, which is an essential part of PHV evaluation with CT, could be visualized using the cine mode with a mean image quality of 3.9±1.4. The use of three subsequent acquisitions offers a wide range of diagnostic possibilities and also provides essential information for planning possible re-interventions. Although the additional acquisition phases may not necessarily change the diagnosis per se, they may be helpful in interpretation and increase confidence. This, however, is difficult to quantify and therefore not included quantitatively in this study. The first, non-enhanced, scan shows the presence and extent of (peri)valvular calcifications. In case of endocarditis, calcifications in pseudoaneurysms around the aortic root allow discrimination between a chronic instead of an active endocarditis, as was the case in one of our patients. Moreover, this non-enhanced scan can identify sutures with polytetrafluorethylene felt pledgets, which were used in eight (19 %) of our patients. These pledgets can be isodense to contrast and mimic paravalvular leakage on CTA, thus a non-enhanced scan can be helpful to differentiate between the two (Fig. 6) [12]. The goal of the second CTA acquisition is to evaluate valve position and valve dynamics as well as to look for possible leakage, obstruction, pannus tissue, thrombus or signs of endocarditis. We used prospective ECG-triggering with three stacks for our CTA acquisition, which lowered the dose by more than 20 % in comparison to retrospective ECG-triggering at the cost of a short lack of data in the first 100 ms after the R-peak of the cardiac cycle [6–8]. By setting the scan length to exactly 14 cm, three stacks were obtained with maximum individual stack coverage to ensure images were without stack artefacts at the level of the valve, as shown by our results (only two of 44 CTAs had stack artefacts, none of them affecting the valve). One might consider scanning a single stack only for the valve. However, the advantage of using three stacks is that the coronary arteries are completely visualized, which may allow an additional coronary angiography to be omitted in case of re-operation. Furthermore, it gives a good overview of the left ventricular outflow tract. In this acquisition, we used an ECG-trigger based on absolute millisecond (ms) time instead of a relative (%) RR-trigger. A ‘scan-on–scan-off’ padding setting from 0–1,500 ms with 50–500 ms ECG-pulsing resulted in a scan acquisition window that was guaranteed to provide the fastest onset of radiation after an R-peak (minimum delay is ±100 ms, which is 10 % at 60 bpm). Radiation was maintained for 1,500 ms (which is a full heart cycle at 40 bpm) or cut off when the next R-peak occurred. This meant that regardless of the heart rate, a full heart cycle (starting from 100 ms) was guaranteed, which makes this protocol very robust and easy to perform. The higher dose at 50–500 ms guaranteed high quality systolic images on which pannus, thrombus and leakage can be evaluated. In diastole, dose was reduced to 20 % (with higher noise levels), which is tolerable since this phase is only needed for evaluation of the leaflet motion and confirmation of pathology detected in systole. Moreover, prospective triggering reduced prosthetic heart valve-induced artefacts in comparison with retrospective ECG-gating [9]. Further development of this protocol could include automatic kV selection for this CTA acquisition in patients with a PHV with a limited amount of metallic components to further lower radiation dose.

**Fig. 7:**
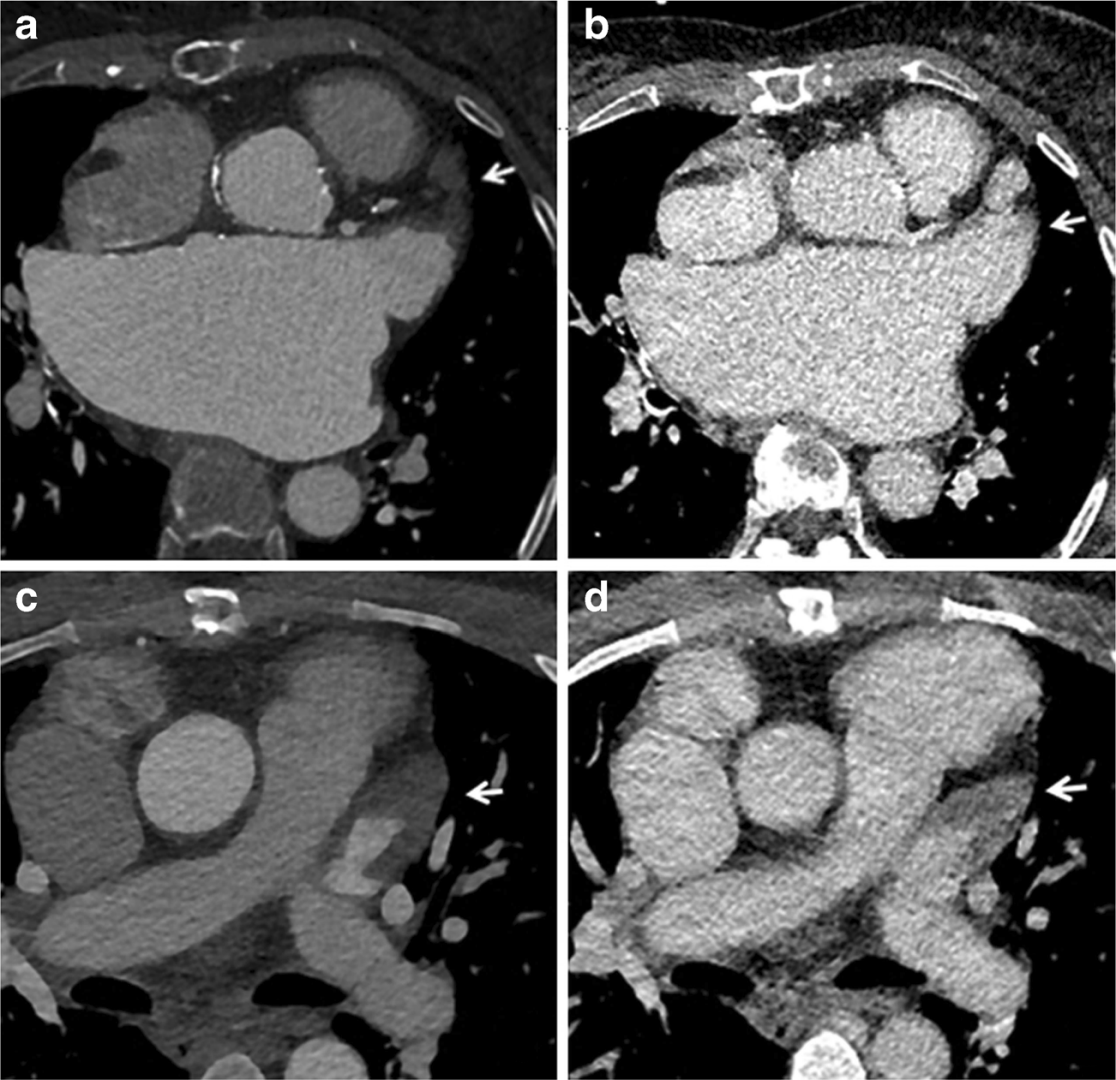
Advantage of a delayed acquisition: differentiating thrombus from flow artefacts. One of the advantages of the delayed scan acquisition is the ability to better depict thrombus and differentiate thrombus from flow artefacts, since patients with a prosthetic heart valve (PHV) often have arrhythmias. In the first patient (**A-B**), the arterial CT angiography (CTA) acquisition (**A**) depicts a filling defect in the left atrial appendage (arrow). The delayed scan (**B**) shows that it involved a flow artefact with good filling of the left atrial appendage in this phase (arrows). Thrombus could thus be excluded. In the second patient (**C-D**), the arterial CTA acquisition (**C**) also depicts a filling defect in the left atrial appendage (arrow). However, in this case, the delayed scan (**D**) confirms the presence of a thrombus (arrow)

The delayed scan comprised the entire chest and thus the full thoracic aorta, at a relatively low radiation cost. The advantages of this ‘add-on’ scan may be the identification of the extent of disease beyond valves, emphasizing infectious attenuation, better depicting thrombus and differentiating thrombus from flow artefacts (e.g. in the left atrial appendage, since patients with a PHV often have arrhythmias). In five patients (12 %) we could thus differentiate between thrombus and flow artefacts (Fig. 7). Besides optimization of CT acquisition parameters, another way to achieve dose reduction is to optimize CT image reconstruction through iterative reconstruction techniques. This alternative image reconstruction method allows imaging at a lower radiation-dose with similar noise levels and image quality compared to routine dose filtered back-projection (FBP), thus reducing dose without compromising image quality [13].

Although a mean radiation dose of 8.3±3.0 mSv for all three acquisitions combined is considerably lower than reported previously [6–8], it is not minimal. However, one must take into account that this patient population was relatively old and represents a population that often has serious pathology with relatively high morbidity and mortality, e.g. due to endocarditis, with a genuine possibility of needing a high-risk reoperation. This imposes a good balance between lowering the radiation dose as much as possible but still gathering all the information needed for good clinical decision making and preoperative planning. In follow-up CT examinations, and depending on the clinical question, the non-enhanced or delayed scan can be omitted and the CTA can be obtained using only one stack through the valve, where relevant. As underlined in several recent studies, another additional benefit of CT is the power to detect leaflet thickening (most likely due to thrombus formation) of biological and transcatheter PHVs that was not detected by TTE [14–16]. The possible addition of CT in the routine work-up early after PHV implantation also necessitates lowering the radiation dose as much as possible.

Our study had some limitations. In this descriptive study of a new CT protocol for the evaluation of PHVs, no comparison with other acquisition protocols in the same or a similar group of patients was made. Furthermore, albeit irrelevant to our main outcome of image quality, no comparison with a reference standard was made regarding pathological findings.

## Conclusion

A novel comprehensive CT image acquisition protocol is presented that allows for both dynamic reconstructions to assess PHV leaflet motion and high-resolution anatomical images with good image quality, using third-generation dual-source CT and iterative reconstruction techniques. The moderate radiation dose of 8.3 ±3.0 mSv for this three-phase acquisition is – albeit not minimal – substantially lower (> 20 % reduction) than previous (usually retrospectively ECG-gated) single-phase image acquisition techniques and can be considered acceptable when seen in relation to the pathology, the risks and possible re-intervention in this patient population.

## **Abbreviations**

CTA: Computed tomography angiography
DSCT: Dual-source computed tomography
IR: Iterative reconstruction
MDCT: Multidetector computed tomography
PHV: Prosthetic heart valve
PVL: Paravalvular leakage
TEE: Transoesophageal echocardiography
TTE: Transthoracic echocardiography
